# Basis properties of the *p*, *q*-sine functions

**DOI:** 10.1098/rspa.2014.0642

**Published:** 2015-02-08

**Authors:** Lyonell Boulton, Gabriel J. Lord

**Affiliations:** Department of Mathematics and Maxwell Institute for Mathematical Sciences, Heriot-Watt University, Edinburgh EH14 4AS, UK

**Keywords:** Riesz basis, Schauder basis, generalized trigonometric functions

## Abstract

We improve the currently known thresholds for basisness of the family of periodically dilated *p*,*q*-sine functions. Our findings rely on a Beurling decomposition of the corresponding change of coordinates in terms of shift operators of infinite multiplicity. We also determine refined bounds on the Riesz constant associated with this family. These results seal mathematical gaps in the existing literature on the subject.

## 1. Introduction

Let *p*,*q*>1. Let *F*_*p*,*q*_:[0,1]→[0,*π*_*p*,*q*_/2] be the integral

$$F_{p,q}(y)=\int_{0}^{y}\frac{dx}{{\left(1-x^{q}\right)}^{1/p}},$$

where *π*_*p*,*q*_=2*F*_*p*,*q*_(1). The *p*,*q*-*sine functions*, $\sin_{p,q}:R⟶[-1,1],$ are defined to be the inverses of *F*_*p*,*q*_,

$$\sin_{p,q}(x)=F_{p,q}^{-1}(x)\;\text{for all}\;x\in \left[0,\frac{\pi_{p,q}}{2}\right]$$

extended to R by the rules

$$\begin{array}{rl} \sin_{p,q}(-x) & =-\sin_{p,q}(x)\;\text{and}\\ \sin_{p,q}\left(\frac{\pi_{p,q}}{2}-x\right) & =\sin_{p,q}\left(\frac{\pi_{p,q}}{2}+x\right), \end{array}$$

which make them periodic, continuous, odd with respect to 0 and even with respect to *π*_*p*,*q*_/2. These are natural generalizations of the sine function, indeed,

$$\sin_{2,2}(x)=\sin(x)\;\text{and}\;\pi_{2,2}=\pi ,$$

and they are known to share a number of remarkable properties with their classical counterpart [1,2].

Among these properties lie the fundamental question of completeness and linear independence of the family $S={\left\{s_{n}\right\}}_{n=1}^{\infty }$, where $s_{n}(x)=\sin_{p,q}(\pi_{p,q}nx)$. This question has received some attention recently [2–5], with a particular emphasis on the case *p*=*q*. In the latter instance, S is the set of eigenfunctions of the generalized eigenvalue problem for the one-dimensional *p*-Laplacian subjected to Dirichlet boundary conditions [6,7], which is known to be of relevance in the theory of slow/fast diffusion processes, [8]. See also the related papers [9,10].

Set $e_{n}(x)=\sqrt{2}\sin(n\pi x)$, so that ${\left\{e_{n}\right\}}_{n=1}^{\infty }$ is a Schauder basis of the Banach space *L*^*r*^≡*L*^*r*^(0,1) for all *r*>1. The family S is also a Schauder basis of *L*^*r*^ if and only if the corresponding *change of coordinates map*, *A*:*e*_*n*_↦*s*_*n*_, extends to a linear homeomorphism of *L*^*r*^. The Fourier coefficients of *s*_*n*_(*x*) associated with *e*_*k*_ obey the relation

$$\begin{array}{rl} {\hat{s}}_{n}(k) & =\int_{0}^{1}s_{1}(nx)e_{k}(x)\;dx\\ & =\sum_{m=1}^{\infty }{\hat{s}}_{1}(m)\int_{0}^{1}e_{mn}(x)e_{k}(x)\;dx=\left\{ \begin{array}{ll} {\hat{s}}_{1}(m) & \text{if }mn=k\text{ for some}m\in N\text{\}}\\ 0 & \text{otherwise.} \end{array}\right. \end{array}$$

For j∈N, let

$$a_{j}\equiv a_{j}(p,q)={\hat{s}}_{1}(j)=\sqrt{2}\int_{0}^{1}\sin_{p,q}(\pi_{p,q}x)\sin(j\pi x)\;dx$$

(note that *a*_*j*_=0 for *j*≡_2_0) and let *M*_*j*_ be the linear isometry such that *M*_*j*_*e*_*k*_=*e*_*jk*_. Then,

$$Ae_{n}=s_{n}=\sum_{k=1}^{\infty }{\hat{s}}_{n}(k)e_{k}=\sum_{j=1}^{\infty }{\hat{s}}_{1}(j)e_{jn}=\left(\sum_{j=1}^{\infty }a_{j}M_{j}\right)e_{n},$$

so that the change of coordinates takes the form

1.1 $$A=\sum_{j=1}^{\infty }a_{j}M_{j}.$$

Notions of ‘nearness’ between bases of Banach spaces are known to play a fundamental role in classical mathematical analysis [11], pp. 265–266, [12], §I.9 or [13], p. 71. Unfortunately, the expansion (1.1) strongly suggests that S is not globally ‘near’ ${\left\{e_{n}\right\}}_{n=1}^{\infty }$, for example, in the Krein–Lyusternik or the Paley–Wiener sense [12], p. 106. Therefore, classical arguments, such as those involving the Paley–Wiener stability theorem, are unlikely to be directly applicable in the present context.

In fact, more rudimentary methods can be invoked in order to examine the invertibility of the change of coordinates map. From (1.1), it follows that

1.2 $$\sum_{j=3}^{\infty }|a_{j}|<|a_{1}|\Rightarrow \left\{ \begin{array}{l} A,A^{-1}\in B(L^{r})\\ ∥A∥\;∥A^{-1}∥\leq \frac{\sum_{j=1}^{\infty }|a_{j}|}{|a_{1}|-\sum_{j=3}^{\infty }|a_{j}|}. \end{array}\right.$$

In Binding *et al.* [5], it was claimed that the left-hand side of (1.2) held true for all *p*=*q*≥*p*_1_, where *p*_1_ was determined to lie in the segment $\left(1,\frac{12}{11}\right)$. Hence, S would be a Schauder basis, whenever $p=q\in (p_{1},\infty )$.

Further developments in this respect were recently reported by Bushell & Edmunds [4]. These authors cleverly fixed a gap originally published in [5], lemma 5 and observed that, as the left-hand side of (1.2) ceases to hold true whenever

1.3 $$a_{1}=\sum_{j=3}^{\infty }a_{j},$$

the argument will break for *p*=*q* near *p*_2_≈1.043989. Therefore, the basisness question for S should be tackled by different means in the regime *p*,*q*→1.

More recently, Edmunds *et al.* [3] employed (1.2) in order to show invertibility of *A* for general pairs (*p*,*q*), as long as

1.4 $$\pi_{p,q}<\frac{16}{\pi^{2}-8}.$$

Because (1.4) is guaranteed whenever

1.5 $$\frac{p}{q(p-1)}<\frac{4}{\pi^{2}-8},$$

this allows *q*→1 for *p*>4/(12−*π*^2^). However, note that a direct substitution of *p*=*q* in (1.5) only leads to the suboptimal condition *p*>*π*^2^/4−1≈1.467401.

In §2, we show that the family S is *ω*-*linearly independent* for all *p*,*q*>1, see theorem 2.1. In §5, we establish conditions ensuring that *A* is a homeomorphism of *L*^2^ in a neighbourhood of the region in the (*p*,*q*)-plane where

$$\sum_{j=3}^{\infty }|a_{j}|=a_{1},$$

see theorem 5.1 and also corollary 6.2. For this purpose, in §4, we find two further criteria which generalize (1.2) in the Hilbert space setting, see corollaries 4.3 and 4.4. In this case, the *Riesz constant*,

$$r(S)=∥A∥\;∥A^{-1}∥,$$

characterizes how S deviates from being an orthonormal basis. These new statements yield upper bounds for r(S), which improve upon those obtained from the right-hand side of (1.2), even when the latter is applicable.

The formulation of the alternatives to (1.2) presented below relies crucially on work developed in §3. From lemma 3.1, we compute explicitly the Wold decomposition of the isometries *M*_*j*_: they turn out to be shifts of infinite multiplicity. Hence, we can extract from the expansion (1.1) suitable components which are Toeplitz operators of scalar type acting on appropriate Hardy spaces. As the theory becomes quite technical for the case *r*≠2 and all the estimates analogous to those reported below would involve a dependence on the parameter *r*, we have chosen to restrict our attention with regards to these improvements only to the already interesting Hilbert space setting.

Section 6 is concerned with particular details of the case of equal indices *p*=*q*, and it involves results on both the general case *r*>1 and the specific case *r*=2. Rather curiously, we have found another gap which renders incomplete the proof of invertibility of *A* for *p*_1_<*p*<2 originally published in [5]. See remark 6.3. Moreover, the application of Bushell & Edmunds [4], theorem 4.5 only gets to a *basisness threshold* of ${\tilde{p}}_{1}\approx 1.198236>\frac{12}{11}$, where ${\tilde{p}}_{1}$ is defined by the identity

1.6 $$\pi_{{\tilde{p}}_{1},{\tilde{p}}_{1}}=\frac{2\pi^{2}}{\pi^{2}-8}.$$

See also [2], remark 2.1. In theorem 6.5, we show that S is indeed a Schauder basis of *L*^*r*^ for $p=q\in (p_{3},\frac{6}{5}),$ where $p_{3}\approx 1.087063<\frac{12}{11}$, see [14], problem 1. As $\frac{6}{5}>{\tilde{p}}_{1}$, basisness is now guaranteed for all *p*=*q*>*p*_3_ (figure 3).

In §7, we report on our current knowledge of the different thresholds for invertibility of the change of coordinates map, both in the case of equal indices and otherwise. Based on the new criteria found in §4, we formulate a general test of invertibility for *A* which is amenable to analytical and numerical investigation. This test involves finding sharp bounds on the first few coefficients *a*_*k*_(*p*,*q*). See proposition 7.1. For the case of equal indices, this test indicates that S is a Riesz basis of *L*^2^ for *p*=*q*>*p*_6_, where *p*_6_≈1.043917<*p*_2_.

All the numerical quantities reported in this paper are accurate up to the last digit shown, which is rounded to the nearest integer. In the online version of this manuscript,^1^ we have included fully reproducible computer codes which can be employed to verify the calculations reported.

## 2. Linear independence

A family ${\left\{{\tilde{s}}_{n}\right\}}_{n=1}^{\infty }$ in a Banach space is called *ω*-linearly independent [12], p. 50, if

$$\sum_{n=1}^{\infty }f_{n}{\tilde{s}}_{n}=0\;\Rightarrow \;f_{n}=0\;\text{for all }n.$$

**Theorem 2.1**

*For all p,q*>1, *the family* S *is ω-linearly independent in L*^*r*^*. Moreover, if the linear extension of the map A:e*_*n*_*↦s*_*n*_ *is a bounded operator A:L*^2^*→L*^2^*, then*

$${\left(\bar{\mathrm{span}\;S}\right)}^{⊥}=\mathrm{Ker}\;A^{*}.$$

**Proof.**

For the first assertion, we show that Ker(*A*)={0}. Let $f=\sum_{k=1}^{\infty }\;f_{k}e_{k}$ be such that *Af*=0, where the series is convergent in the norm of *L*^*r*^. Then,

$$\sum_{j=1}^{\infty }\left(\sum_{mn=j}f_{m}a_{n}\right)e_{j}=\sum_{jk=1}^{\infty }f_{k}a_{j}e_{jk}=0.$$

Hence,

2.1 $$\sum_{mn=j}f_{m}a_{n}=0\;\forall \;j\in N.$$

We show that all *f*_*j*_=0 by means of a double induction argument.

Suppose that *f*_1_≠0. We prove that all *a*_*k*_=0. Indeed, clearly *a*_1_=0 from (2.1) with *j*=1. Now, assume inductively that *a*_*j*_=0 for all *j*=1,…,*k*−1. From (2.1), for *j*=*k*, we obtain

$$0=f_{1}a_{k}+\sum_{\begin{array}{c} mn=k\\ m\neq 1\;n\neq k \end{array}}f_{m}a_{n}=f_{1}a_{k}.$$

Then, *a*_*k*_=0 for all k∈N. As this would contradict the fact that *A*≠0, necessarily *f*_1_=0.

Suppose now inductively that *f*_1_,…,*f*_*l*−1_=0 and *f*_*l*_≠0. We prove that again all *a*_*k*_=0. First, *a*_1_=0 from (2.1) with *j*=*l*, because

$$0=f_{l}a_{1}+\sum_{\begin{array}{c} mn=l\\ m\neq l\;n\neq 1 \end{array}}f_{m}a_{n}=f_{l}a_{1}.$$

Second, assume by induction that *a*_*j*_=0 for all *j*=1,…,*k*−1. From (2.1) for *j*=*lk*, we obtain

$$0=f_{l}a_{k}+\sum_{\begin{array}{c} mn=lk\\ m\neq l\;n\neq k \end{array}}f_{m}a_{n}=f_{l}a_{k}.$$

The latter equality is a consequence of the fact that, for *mn*=*lk* with *m*≠*l* and *n*≠*k*, either *m*<*l* (indices for the *f*_*m*_) or *n*<*k* (indices for the *a*_*n*_). Hence, *a*_*k*_=0 for all k∈N. As this would again contradict the fact that *A*≠0, necessarily all *f*_*k*_=0, so that *f*=0.

The second assertion is shown as follows. Assume that $A\in B(L^{2})$. If *f*∈*Ker* A*, then 〈*f*,*Ag*〉=0 for all *g*∈*L*^2^, so *f*⊥Ran *A* which, in turns, means that *f*⊥*s*_*n*_ for all n∈N. On the other hand, if the latter holds true for *f*, then *f*⊥*Ae*_*n*_ for all n∈N, so A*f=0, as required. ▪

Therefore, S is a Riesz basis of *L*^2^ if and only if $A\in B(L^{2})$ and *RanA*=*L*^2^. A simple example illustrates how a family of dilated periodic functions can break its property of being a Riesz basis.

**Example 2.2**

Let *α*∈[0,1]. Take

2.2 $$\tilde{s}(x)=\frac{1-\alpha }{\sqrt{2}}\sin(\pi x)+\frac{\alpha }{\sqrt{2}}\sin(3\pi x).$$

By virtue of lemma 4.1, $\tilde{S}={\left\{\tilde{s}(nx)\right\}}_{n=1}^{\infty }$ is a Riesz basis of *L*^2^ if and only if $0\leq \alpha <\frac{1}{2}$. For *α*=1, we have an orthonormal set. However, it is not complete, as it clearly misses the infinite-dimensional subspace *Span*{*e*_*j*_}_*j*≢_3_0_.

## 3. The different components of the change of coordinates map

The fundamental decomposition of *A* given in (1.1) allows us to extract suitable components formed by Toeplitz operators of scalar type [15]. In order to identify these components, we begin by determining the Wold decomposition of the isometries *M*_*j*_, [15,16]. See remark 3.4.

**Lemma 3.1**

*For all* *j*>1, $M_{j}\in B(L^{2})$ *is a shift of infinite multiplicity*.

**Proof.**

Define

$$L_{0}^{j}=\mathrm{span}{\left\{e_{k}\right\}}_{k{≢}_{j}0}=\mathrm{Ker}(M_{j}^{*})$$

and

$$L_{n}^{j}=M_{j}^{n}L_{0}^{j}\;\text{for}\;n\in N.$$

Then, $L_{n}^{j}\cap L_{m}^{j}=\{0\}$ for *m*≠*n*, $L^{2}=\overset{\infty }{\underset{n=0}{⨁}}L_{n}^{j}$, and $M_{j}:L_{n-1}^{j}⟶L_{n}^{j}$ one-to-one and onto for all n∈N. Therefore, indeed, *M*_*j*_ is a shift of multiplicity $\dim L_{0}^{j}=\infty$. ▪

Let D={|z|<1}. The Hardy spaces of functions in D with values in the Banach space C are denoted below by $H^{\gamma }(D;C)$. Let

$$\tilde{b}(z)=\sum_{k=0}^{\infty }b_{k}z^{k}$$

be a holomorphic function on $\bar{D}$ and fix j∈N∖{1}. Let

$$\tilde{B}\in H^{\infty }(D;B(L_{0}^{j}))\;\text{be given by}\;\tilde{B}(z)=\tilde{b}(z)I.$$

Let the corresponding Toeplitz operator [15], (5-1)

$$T(\tilde{B})\in B(H^{2}(D;L_{0}^{j}))\;\text{be given by}\;T(\tilde{B}):f(z)\mapsto \tilde{B}(z)f(z).$$

Let

3.1 $$B=\sum_{k=0}^{\infty }b_{k}M_{j^{k}}:L^{2}⟶L^{2}.$$

By virtue of lemma 3.1 (see [15], §3.2 and §5.2), there exists an invertible isometry

$$U:L^{2}⟶H^{2}(D;L_{0}^{j})$$

such that $UB=T(\tilde{B})U$. Below, we write

$$M(\tilde{b})=\max_{z\in \bar{D}}|\tilde{b}(z)|\;\text{and}\;m(\tilde{b})=\min_{z\in \bar{D}}|\tilde{b}(z)|.$$

**Theorem 3.2**

*B in* (*3.1*) *is invertible if and only if* $m(\tilde{b})>0$*. Moreover,*

$$∥B∥=M(\tilde{b})\;\text{and}\;∥B^{-1}∥=m{\left(\tilde{b}\right)}^{-1}.$$

**Proof.**

Observe that $T(\tilde{B})$ is scalar analytic in the sense of Rosenblum & Rovnyak [15], §3.9. Because $\tilde{b}$ is holomorphic in $\bar{D}$, then $M(\tilde{b})<\infty$ and

$$∥B∥=∥T(\tilde{B})∥=∥\tilde{B}{∥}_{H^{\infty }(D;B(L_{0}^{j}))}=M(\tilde{b})$$

[15], §4.7 and theorem A(iii).

If $0\notin \tilde{b}(\bar{D})$, then $\tilde{b}{\left(z\right)}^{-1}$ is also holomorphic in $\bar{D}$. The scalar Toeplitz operator $T(\tilde{b})$ is invertible if and only if $m(\tilde{b})>0$. Moreover, [17], §1.5,

$$T{\left(\tilde{b}\right)}^{-1}=T({\tilde{b}}^{-1})\in B(H^{2}(D;C)).$$

The matrix of $T(\tilde{B})$ has the block representation [15], §5.9

$$T(\tilde{B})∼\left[\begin{array}{ccccc} b_{0}I & 0 & 0 & \cdots \\ b_{1}I & b_{0}I & 0 & \cdots \\ b_{2}I & b_{1}I & b_{0}I & \cdots \\ & & \cdots & & \end{array}\right]\;\text{for}\;I\in B(L_{0}^{j}).$$

The matrix associated with $T(\tilde{b})$ has exactly the same scalar form, replacing *I* by 1∈B(C). Then, $T(\tilde{B})$ is invertible if and only if $T(\tilde{b})$ is invertible, and

$$T{\left(\tilde{B}\right)}^{-1}∼\left[\begin{array}{ccccc} b_{0}^{\left(-1\right)}I & 0 & 0 & \cdots \\ b_{1}^{\left(-1\right)}I & b_{0}^{\left(-1\right)}I & 0 & \cdots \\ b_{2}^{\left(-1\right)}I & b_{1}^{\left(-1\right)}I & b_{0}^{\left(-1\right)}I & \cdots \\ & & \cdots & & \end{array}\right]\;\text{for}\;\tilde{b}{\left(z\right)}^{-1}=\sum_{k=0}^{\infty }b_{k}^{\left(-1\right)}z^{k}.$$

Hence,

$$∥B^{-1}∥=∥T{\left(\tilde{B}\right)}^{-1}∥=M({\tilde{b}}^{-1})=m{\left(\tilde{b}\right)}^{-1}.$$

 ▪

**Corollary 3.3**

*Let* *A*=*B*+*C* *for* *B* *as in* (*3.1*). *If* $∥C∥<m(\tilde{b}),$ *then* *A* *is invertible. Moreover*,

3.2 $$∥A∥\leq M(\tilde{b})+∥C∥\;\text{and}\;∥A^{-1}∥\leq \frac{1}{m(\tilde{b})-∥C∥}.$$

**Proof.**

Because *B* is invertible, write *A*=(*I*+*CB*^−1^)*B*. If additionally ∥*CB*^−1^∥<1, then

$$\begin{array}{r} ∥{\left(I+CB^{-1}\right)}^{-1}∥\leq \frac{1}{1-∥C∥\;∥B^{-1}∥}. \end{array}$$

 ▪

**Remark 3.4**

It is possible to characterize the change of coordinates *A* in terms of Dirichlet series, and recover some of the results here and below directly from this characterization. See for example the insightful paper [18] and the complete list of references provided in the addendum [19]. However, the full technology of Dirichlet series is not needed in the present context. A further development in this direction is reported elsewhere.

## 4. Invertibility and bounds on the riesz constant

A proof of (1.2) can be achieved by applying corollary 3.3 assuming that

$$B=a_{1}M_{1}=a_{1}I.$$

Our next goal is to formulate concrete sufficient condition for the invertibility of *A* and corresponding bounds on r(S), which improve upon (1.2), whenever *r*=2. For this purpose, we apply corollary 3.3 assuming that *B* has now the three-term expansion

$$B=a_{1}M_{1}+a_{3}M_{3}+a_{9}M_{9}.$$

Let

T={β<1, β−α+1>0, β+α+1>0}.

Let

$$\begin{array}{rl} R_{1} & =\{|\alpha (\beta +1)|<|4\beta |\}\cap \{\beta >0\}\\ R_{3} & =\{|\alpha (\beta +1)|<|4\beta |\}\cap \{\beta <0\}\\ R_{2} & =\{|\alpha (\beta +1)|\geq |4\beta |\}=R^{2}\setminus (R_{1}\cup R_{3}). \end{array}$$

See figure 1.

**Figure 1.**
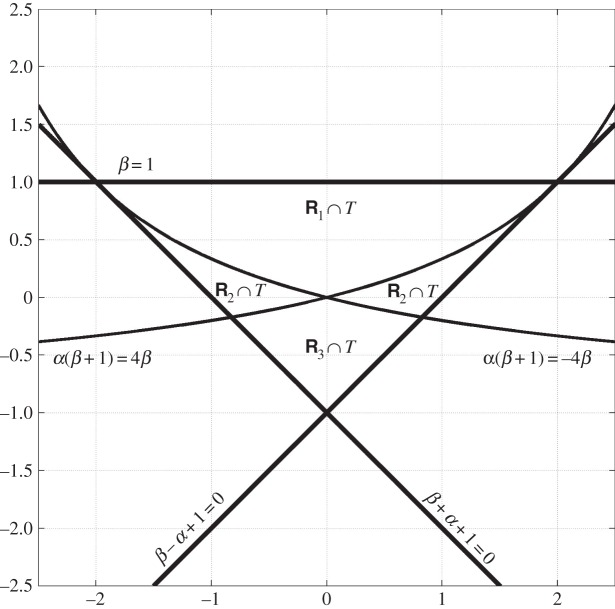
Optimal region of invertibility in lemma 4.1. The horizontal axis is *α* and the vertical axis is *β*.

**Lemma 4.1**

*Let* *r*=2. *Let* α,β∈R. *The operator* *B*=*I*+*αM*_3_+*βM*_9_ *is invertible if and only if* (*α*,*β*)∈*T*. *Moreover*,

$$\left[\begin{array}{c} ∥B∥\\ ∥B^{-1}{∥}^{-1} \end{array}\right]=\left\{ \begin{array}{ll} \left[\begin{array}{c} 1+\beta +|\alpha |\\ (1-\beta )\sqrt{1-\frac{\alpha^{2}}{4\beta }} \end{array}\right] & (\alpha ,\beta )\in R_{1}\cap T\\ \left[\begin{array}{c} 1+\beta +|\alpha |\\ 1+\beta -|\alpha | \end{array}\right] & (\alpha ,\beta )\in R_{2}\cap T\\ \left[\begin{array}{c} (1-\beta )\sqrt{\frac{\alpha^{2}}{4\beta }-1}\\ 1+\beta -|\alpha | \end{array}\right] & (\alpha ,\beta )\in R_{3}\cap T \end{array}\right.$$

**Proof.**

Let $\tilde{b}(z)=1+\alpha z+\beta z^{2}$ be associated with *B* as in §3.

The first assertion is a consequence of the following observation. If *α*^2^−4*β*<0, then $\tilde{b}(z)$ has roots *z*_±_ conjugate with each other and |*z*_±_|≤1 if and only if *β*≥1. Otherwise, $\tilde{b}(z)$ has two real roots. If *α*^2^−4*β*≥0 and *α*≥0, then the smallest in modulus root of $\tilde{b}(z)$ would lie in $\bar{D}$ if and only if *β*−*α*+1≤0. If *α*^2^−4*β*≥0 and *α*<0, then the root of $\tilde{b}(z)$ that is smallest in modulus would lie in $\bar{D}$ if and only if *β*+*α*+1≤0.

For the second assertion, let (*α*,*β*)∈*T* and $b(\theta )=|\tilde{b}(e^{i\theta }){|}^{2}$. By virtue of the maximum principle on $\tilde{b}(z)$ and $1/\tilde{b}(z)$,

$$M{\left(\tilde{b}\right)}^{2}=\max_{-\pi \leq \theta <\pi }b(\theta )\;\text{and}\;m{\left(\tilde{b}\right)}^{2}=\min_{-\pi \leq \theta <\pi }b(\theta ).$$

Because

$$\begin{array}{rl} b(\theta ) & ={\left(1+\alpha \cos(\theta )+\beta \cos(2\theta )\right)}^{2}+{\left(\alpha \sin(\theta )+\beta \sin(2\theta )\right)}^{2}\\ & =1+\alpha^{2}+\beta^{2}+2(\beta +1)\alpha \cos(\theta )+2\beta \cos(2\theta ), \end{array}$$

then *b*′(*θ*)=0 if and only if (α(β+1)+4βcos⁡(θ))sin⁡(θ)=0. For $\sin(\theta_{0})=0$, we obtain *b*(*θ*_0_)=(1+*β*+*α*)^2^ and *b*(*θ*_0_)=(1+*β*−*α*)^2^. For $\cos(\theta_{0})=-\alpha (\beta +1)/4\beta$, we obtain *b*(*θ*_0_)=(1 − *α*^2^/4*β*)(*β*−1)^2^ with the condition |*α*(*β*+1)/4*β*|≤1. By virtue of theorem 3.2, we obtain the claimed statement. ▪

Because $\sin_{p,q}(x)>0$ for all *x*∈(0,*π*_*p*,*q*_), then *a*_1_>0. Below, we substitute *α*=*a*_3_/*a*_1_ and *β*=*a*_9_/*a*_1_, then apply lemma 4.1 appropriately in order to determine the invertibility of *A* whenever pairs (*p*,*q*) lie in different regions of the (*p*,*q*)-plane. For this purpose, we establish the following hierarchy between *a*_1_ and *a*_*j*_ for *j*=3,9, whenever the latter are non-negative.

**Lemma 4.2**

*For* *j*=3 *or* *j*=9, *we have* *a*_*j*_<*a*_1_.

**Proof.**

First, observe that $\sin_{p,q}(\pi_{p,q}x)$ is continuous, it increases for all $x\in (0,\frac{1}{2})$ and it vanishes at *x*=0.

Let *j*=3. Set

$$I_{0}=\int_{0}^{1/4}\sin_{p,q}(\pi_{p,q}x)[\sin(\pi x)-\sin(3\pi x)]\;dx$$

and

$$I_{1}=\int_{1/4}^{1/2}\sin_{p,q}(\pi_{p,q}x)[\sin(\pi x)-\sin(3\pi x)]\;dx.$$

Because

sin⁡(πx)−sin⁡(3πx)=−2sin⁡(πx)cos⁡(2πx),

then *I*_0_<0 and *I*_1_>0. As cos⁡(2πx) is odd with respect to $\frac{1}{4}$ and sin⁡(πx) is increasing in the segment $\left(0,\frac{1}{2}\right)$, then also |*I*_0_|<|*I*_1_|. Hence,

$$a_{1}-a_{3}=2\sqrt{2}(I_{0}+I_{1})>0,$$

ensuring the first statement of the lemma.

Let *j*=9. A straightforward calculation shows that sin⁡(πx)=sin⁡(9πx) if and only if, either sin⁡(4πx)=0 or cos⁡(4πx)cos⁡(πx)=sin⁡(4πx)sin⁡(πx). Thus, sin⁡(πx)−sin⁡(9πx) has exactly five zeros in the segment $\left[0,\frac{1}{2}\right]$ located at

$$x_{0}=0,\;x_{1}=\frac{1}{10},\;x_{4}=\frac{1}{4},\;x_{5}=\frac{3}{10}\;\text{and}\;x_{8}=\frac{1}{2}.$$

Set

$$x_{2}=\frac{1}{9},\;x_{3}=\frac{19}{90},\;x_{6}=\frac{13}{36}\;\text{and}\;x_{7}=\frac{37}{90},$$

and

$$I_{k}=\int_{x_{k}}^{x_{k+1}}\sin_{p,q}(\pi_{p,q}x)[\sin(\pi x)-\sin(9\pi x)]\;dx.$$

Then, *I*_*k*_<0 for *k*=0,4 and *I*_*k*_>0 for *k*=1,2,3,5,6,7. Because

$$\sin(9\pi x)-\sin(\pi x)<\sin(\pi (x+\frac{1}{9}))-\sin(9\pi (x+\frac{1}{9}))$$

for all $x\in (0,\frac{1}{2})$, then

$$|I_{0}|<|I_{2}|\;\text{and}\;|I_{4}|<|I_{6}|.$$

Hence,

$$\begin{array}{r} a_{1}-a_{9}=2\sqrt{2}\sum_{k=0}^{7}I_{k}>2\sqrt{2}(I_{1}+I_{3}+I_{5}+I_{7})>0. \end{array}$$

 ▪

Corollaries 4.3 and 4.4 are consequences of corollary 3.3 and lemma 4.1, and are among the main results of this paper.

**Corollary 4.3**

4.1 $$\begin{array}{rl} & \left(\frac{a_{3}}{a_{1}},\frac{a_{9}}{a_{1}}\right)\in R_{2}\cap T\\ & \sum_{j\notin \{1,9\}}^{\infty }|a_{j}|<a_{1}+a_{9} \end{array}\}\;\Rightarrow \;\left\{ \begin{array}{rl} & A,A^{-1}\in B(L^{2})\\ & r(S)\leq \frac{\sum_{j=1}^{\infty }|a_{j}|}{a_{1}+a_{9}-\sum_{j\notin \{1,9\}}^{\infty }|a_{j}|}. \end{array}\right.$$

**Proof.**

Let *A*=*B*+*C*, where

$$B=a_{1}I+a_{3}M_{3}+a_{9}M_{9}\;\text{and}\;C=\sum_{j\notin \{1,3,9\}}^{\infty }a_{j}M_{j}.$$

The top on the left-hand side of (4.1) and the fact that *a*_1_>0 imply

$$∥B^{-1}{∥}^{-1}=a_{1}-|a_{3}|+a_{9}.$$

Thus, the bottom on the left-hand side of (4.1) yields

$$∥C∥\leq \sum_{j\notin \{1,3,9\}}^{\infty }|a_{j}|<∥B^{-1}{∥}^{-1},$$

so, indeed, *A* is invertible. The estimate on the Riesz constant is deduced from the triangle inequality. ▪

Because *a*_1_>0, (4.1) supersedes (1.2), only when the pair (*p*,*q*) is such that *a*_9_>0. From this corollary, we see below that the change of coordinates is invertible in a neighbourhood of the threshold set by the condition (1.3). See proposition 7.1 and figures 3 and 4].

**Corollary 4.4**

4.2 (a3a1,a9a1)∈R1∩T∑j∉{1,3,9}∞|aj|<(a1−a9)(1−a324a1a9)1/2}⇒{(a3a1,a9a1)A,A−1∈B(L2)∑j∉{1,3,9}∞r(S)≤∑j=1∞|aj|(a1−a9)(1−a32/4a1a9)1/2−∑j∉{1,3,9}∞|aj|.

**Proof.**

The proof is similar to that of corollary 4.3. ▪

We see in the following that corollary 4.3 is slightly more useful than corollary 4.4 in the context of the dilated *p*,*q*-sine functions. However, the latter is needed in the proof of the main theorem 5.1.

It is of course natural to ask what consequences can be derived from the other statement in lemma 4.1. For

$$\left(\frac{a_{3}}{a_{1}},\frac{a_{9}}{a_{1}}\right)\in R_{3}\cap T,$$

we have ∥*B*^−1^∥^−1^=*a*_1_−|*a*_3_|−|*a*_9_|. Hence, the same argument as in the proofs of corollaries 4.3 and 4.4 would reduce to (1.2), and in this case, there is no improvement.

## 5. Riesz basis properties beyond the applicability of (1.2)

Our first goal in this section is to establish that the change of coordinates map associated with the family S is invertible beyond the region of applicability of (1.2). We begin by recalling a calculation which was performed in the proof of [3], proposition 4.1 and which will be invoked several times below. Let *a*(*t*) be the inverse function of $\sin_{p,q}^{\prime }(\pi_{p,q}t)$. Then,

5.1 $$a_{j}(p,q)=-\frac{2\sqrt{2}\pi_{p,q}}{j^{2}\pi^{2}}\int_{0}^{1}\sin\left(\frac{j\pi }{\pi_{p,q}}a(t)\right)dt.$$

Indeed, integrating by parts twice and changing the variable of integration to

$$t=\sin_{p,q}^{\prime }(\pi_{p,q}x)$$

yields

$$\begin{array}{rl} a_{j}(p,q) & =\sqrt{2}\int_{0}^{1}\sin_{p,q}(\pi_{p,q}x)\sin(j\pi x)\;dx\\ & =2\sqrt{2}\int_{0}^{1/2}\sin_{p,q}(\pi_{p,q}x)\sin(j\pi x)\;dx\\ & =\frac{2\sqrt{2}\pi_{p,q}}{j\pi }\int_{0}^{1/2}\sin_{p,q}^{\prime }(\pi_{p,q}x)\cos(j\pi x)\;dx\\ & =-\frac{2\sqrt{2}\pi_{p,q}}{j^{2}\pi^{2}}\int_{0}^{1/2}{\left[\sin_{p,q}^{\prime }(\pi_{p,q}x)\right]}^{\prime }\sin(j\pi x)\;dx\\ & =-\frac{2\sqrt{2}\pi_{p,q}}{j^{2}\pi^{2}}\int_{0}^{1}\sin\left(\frac{j\pi }{\pi_{p,q}}a(t)\right)dt. \end{array}$$

**Theorem 5.1**

*Let r=2. Suppose that the pair* $\left(\tilde{p},\tilde{q}\right)$ *is such that the following two conditions are satisfied*

(a) $a_{3}(\tilde{p},\tilde{q}),\;a_{9}(\tilde{p},\tilde{q})>0$
(b) $\sum_{j=3}^{\infty }|a_{j}(\tilde{p},\tilde{q})|=a_{1}(\tilde{p},\tilde{q})$.

*Then, there exists a neighbourhood* $(\tilde{p},\tilde{q})\in N\subset {\left(1,\infty \right)}^{2},$ *such that the change of coordinates A is invertible for all* (p,q)∈N.

**Proof.**

From the dominated convergence theorem, it follows that each *a*_*j*_(*p*,*q*) is a continuous function of the parameters *p* and *q*. Therefore, by virtue of (5.1) and a further application of the dominated convergence theorem, $\sum_{j\in F}|a_{j}|$ is also continuous in the parameters *p* and *q*. Here, F can be any fixed set of indices, but below in this proof we need to consider only F=N∖{1,9} for the first possibility and F=N∖{1,3,9} for the second possibility.

Write ${\tilde{a}}_{j}=a_{j}(\tilde{p},\tilde{q})$. The hypothesis implies $({\tilde{a}}_{3}/{\tilde{a}}_{1},{\tilde{a}}_{9}/{\tilde{a}}_{1})\in T$, because

$$0<\frac{{\tilde{a}}_{3}}{{\tilde{a}}_{1}}+\frac{{\tilde{a}}_{9}}{{\tilde{a}}_{1}}<1.$$

Therefore,

5.2 $$\left(\frac{a_{3}}{a_{1}},\frac{a_{9}}{a_{1}}\right)\in T\cap {\left(0,1\right)}^{2}\;\forall \;(p,q)\in N_{1}$$

for a suitable neighbourhood $(\tilde{p},\tilde{q})\in N_{1}\subset {\left(1,\infty \right)}^{2}$. Two possibilities are now in place.

 *First possibility* $({\tilde{a}}_{3}/{\tilde{a}}_{1},{\tilde{a}}_{9}/{\tilde{a}}_{1})\in R_{2}\cap T$. Note that $\sum_{j\notin \{1,9\}}|{\tilde{a}}_{j}|<{\tilde{a}}_{1}+{\tilde{a}}_{9}$ is an immediate consequence of (a) and (b). By continuity of all quantities involved, there exists a neighbourhood $(\tilde{p},\tilde{q})\in N_{2}\subset {\left(1,\infty \right)}^{2}$ such that the left-hand side and hence the right-hand side of (4.1) hold true for all $(p,q)\in N_{2}$.

 *Second possibility* $({\tilde{a}}_{3}/{\tilde{a}}_{1},{\tilde{a}}_{9}/{\tilde{a}}_{1})\in R_{1}\cap T$. Substitute $\alpha ={\tilde{a}}_{3}/{\tilde{a}}_{1}$ and $\beta ={\tilde{a}}_{9}/{\tilde{a}}_{1}$. If (*α*,*β*)∈*R*_1_∩(0,1)^2^, then

5.3 $$1-\beta -\alpha <(1-\beta )\sqrt{1-\frac{\alpha^{2}}{4\beta }}.$$

Indeed, the conditions on *α* and *β* give

0<α,  β<1,  α(β+1)<4βandα+β<1.

As *β*>*α*/(4−*α*),

$$\sqrt{1-\frac{\alpha^{2}}{4\beta }}>\sqrt{\frac{4-4\alpha +\alpha^{2}}{4}}=1-\frac{\alpha }{2}.$$

Thus,

$$(1-\beta )\sqrt{1-\frac{\alpha^{2}}{4\beta }}>(1-\beta )\left(1-\frac{\alpha }{2}\right)=1-\beta -\frac{\alpha }{2}+\frac{\alpha \beta }{2}>1-\beta -\alpha$$

which is (5.3). Hence,

$$\sum_{j\notin \{1,3,9\}}^{\infty }|{\tilde{a}}_{j}|=({\tilde{a}}_{1}-{\tilde{a}}_{9}-{\tilde{a}}_{3})<({\tilde{a}}_{1}-{\tilde{a}}_{9})\sqrt{1-\frac{{\tilde{a}}_{3}^{2}}{4{\tilde{a}}_{1}{\tilde{a}}_{9}}}.$$

Thus, once again by continuity of all quantities involved, there exists a neighbourhood $(\tilde{p},\tilde{q})\in N_{3}\subset {\left(1,\infty \right)}^{2}$ such that the left-hand side and hence the right-hand side of (4.2) hold true for all $(p,q)\in N_{3}$.

The conclusion follows by defining either $N=N_{1}\cap N_{2}$ or $N=N_{1}\cap N_{3}$. ▪

We now examine other further consequences of the corollaries 4.3 and 4.4.

**Theorem 5.2**

*Any of the following conditions ensure the invertibility of the change of coordinates map A:L*^*r*^*→L*^*r*^.

(a) (*r*>1):
  5.4 $$\frac{\pi_{p,q}}{a_{1}}<\frac{2\sqrt{2}\pi^{2}}{\pi^{2}-8}.$$
(b) (*r*=2): *a*_3_>0, *a*_9_>0, *a*_3_(*a*_1_+*a*_9_)≥4*a*_9_*a*_1_ *and*
  $$\frac{\pi_{p,q}}{a_{1}+a_{9}}<\frac{\pi^{2}}{(\pi^{2}/8-82/81)2\sqrt{2}}.$$
(c) (*r*=2): *a*_3_>0, *a*_9_>0, *a*_3_(*a*_1_+*a*_9_)<4*a*_9_*a*_1_ *and*
  $$\frac{\pi_{p,q}}{(a_{1}-a_{9}){\left(1-a_{3}^{2}/4a_{1}a_{9}\right)}^{1/2}}<\frac{\pi^{2}}{(\pi^{2}/8-91/81)2\sqrt{2}}.$$

**Proof.**

From (5.1), it follows that

5.5 $$\sum_{j\notin \{1\}}|a_{j}|\leq \frac{2\sqrt{2}\pi_{p,q}}{\pi^{2}}\left(\frac{\pi^{2}}{8}-1\right).$$

Hence, the condition (a) implies that the hypothesis (1.2) is satisfied.

By virtue of lemma 4.2, it is guaranteed that

$$\left(\frac{a_{3}}{a_{1}},\frac{a_{9}}{a_{1}}\right)\in {\left(0,1\right)}^{2}\subset T$$

in the settings of (b) or (c). From (5.1), it also follows that

5.6 $$\sum_{j\notin \{1,9\}}|a_{j}|\leq \frac{2\sqrt{2}\pi_{p,q}}{\pi^{2}}\left(\frac{\pi^{2}}{8}-\frac{82}{81}\right)$$

and that

5.7 $$\sum_{j\notin \{1,3,9\}}|a_{j}|\leq \frac{2\sqrt{2}\pi_{p,q}}{\pi^{2}}\left(\frac{\pi^{2}}{8}-\frac{91}{81}\right).$$

Combining each one of these assertions with (4.1) and (4.2), respectively, immediately leads to the claimed statement. ▪

We recover [3], corollary 4.3 from the part (a) of this theorem by observing that for all *p*,*q*>1,

$$a_{1}\geq 2\sqrt{2}\int_{0}^{1/2}2x\sin(\pi x)\;dx=\frac{4\sqrt{2}}{\pi^{2}}.$$

In fact, for (*p*,*q*)∈(1,2)^2^, the better estimate

$$a_{1}\geq 2\sqrt{2}\int_{0}^{1}\sin^{2}(\pi x)\;dx=\frac{\sqrt{2}}{2},$$

ensures invertibility of *A* for all *r*>1 whenever

5.8 $$\pi_{p,q}<\frac{2\pi^{2}}{\pi^{2}-8}.$$

See figures 4 and 5.

## 6. The case of equal indices

We now consider in closer detail the particular case *p*=*q*<2. Our analysis requires setting various sharp upper and lower bounds on the coefficients *a*_*j*_(*p*,*p*) for *j*=1,3,5,7,9. This is our first goal.

**Lemma 6.1**

(a) *a*_3_(*p*,*p*)>0 *for all* $1<p\leq \frac{4}{3}$.
(b) *a*_5_(*p*,*p*)>0 *for all* $1<p\leq \frac{6}{5}$.
(c) *a*_7_(*p*,*p*)>0 *for all* $1<p\leq \frac{6}{5}$.
(d) *a*_9_(*p*,*p*)>0 *for all* $1<p\leq \frac{12}{11}$.

**Proof.**

All the stated bounds are determined by integrating a suitable approximation of $\sin_{p,p}(\pi_{p,p}x)$. Each one requires a different set of quadrature points, but the general structure of the arguments in all cases is similar. Without further mention, in the following, we repeatedly use the fact that in terms of hypergeometric functions,

$$\sin_{p,q}^{-1}(y)=\int_{0}^{y}\frac{dx}{{\left(1-x^{q}\right)}^{1/p}}=y\;{\;}_{2}\text{F}{\;}_{1}\left(\frac{1}{p},\frac{1}{q};\frac{1}{q}+1;y^{q}\right)\;\forall \;y\in [0,1].$$

 *Bound* (a). Let

$${\left\{x_{j}\right\}}_{j=0}^{3}=\left\{0,\frac{1}{6},\frac{1}{3},\frac{1}{2}\right\}\;\text{and}\;{\left\{y_{j}\right\}}_{j=0}^{3}=\left\{0,\frac{3}{4},\frac{\sqrt{3}}{2},1\right\}.$$

For *x*∈[*x*_*j*_,*x*_*j*+1_), let

$$\ell_{j}(x)=\frac{y_{j+1}-y_{j}}{x_{j+1}-x_{j}}(x-x_{j})+y_{j}\;\text{for }j=0,1\;\text{and}\;\ell_{2}(x)=1$$

(figure 2). Because

$$\begin{array}{rl} \sin_{4/3,4/3}^{-1}(y_{1}) & =\left(\frac{3}{4}\right){\;}_{2}\text{F}{\;}_{1}\left(\frac{3}{4},\frac{3}{4};\frac{7}{4};{\left(\frac{3}{4}\right)}^{4/3}\right)\\ & <\frac{105}{100}<\frac{110}{100}<\frac{\pi \sqrt{2}}{4}=\frac{\pi_{4/3,4/3}}{6} \end{array}$$

and $\sin_{p,p}(t)$ is an increasing function of *t*∈(0,*π*_*p*,*p*_/2), then

$$\sin_{4/3,4/3}(\pi_{4/3,4/3}x_{1})>y_{1}.$$

According to Bushell & Edmunds [4], corollary 4.4,^2^ $\sin_{p,p}(\pi_{p,p}x)$ increases as *p* decreases for any fixed *x*∈(0,1). Let *p* be as in the hypothesis. Then

$$\sin_{p,p}(\pi_{p,p}x_{1})>y_{1}$$

and similarly

$$\sin_{p,p}(\pi_{p,p}x_{2})>\sin_{2,2}(\pi_{2,2}x_{2})=y_{2}.$$

By virtue of Binding *et al*. [5], lemma 3, the function $\sin_{p,p}(t)$ is strictly concave for *t*∈(0,*π*_*p*,*p*_/2). Then, in fact,

$$\begin{array}{rl} \sin_{p,p}(\pi_{p,p}x)>\ell_{0}(x) & =\frac{9}{2}x\;\forall \;x\in (x_{0},x_{1})\\ \sin_{p,p}(\pi_{p,p}x)>\ell_{1}(x) & =\left(3\sqrt{3}-\frac{9}{2}\right)x+\frac{3-\sqrt{3}}{2}\;\forall \;x\in (x_{1},x_{2}). \end{array}$$

Let

$$I_{j}=2\sqrt{2}\int_{x_{j}}^{x_{j+1}}\ell_{j}(x)\sin(3\pi x)\;dx.$$

Because sin⁡(3πx)≤0 for $x\in (\frac{1}{3},\frac{1}{2})$ and $|\sin_{p,p}(\pi_{p,p}x)|\leq 1$,

$$\begin{array}{rl} a_{3}(p,p) & =2\sqrt{2}\int_{0}^{1/2}\sin_{p,p}(\pi_{p,p}x)\sin(3\pi x)\;dx\\ & >I_{0}+I_{1}+I_{2}\\ & =2\sqrt{2}\left(\frac{1}{2\pi^{2}}+\frac{(\pi -2)\sqrt{3}+3}{6\pi^{2}}-\frac{1}{3\pi }\right)>0. \end{array}$$

 *Bound* (b). Note that

$$\pi_{6/5,6/5}=\frac{10\pi }{3}.$$

Set

$${\left\{x_{j}\right\}}_{j=0}^{4}=\left\{0,\frac{1}{10},\frac{1}{5},\frac{2}{5},\frac{1}{2}\right\}\;\text{and}\;{\left\{y_{j}\right\}}_{j=0}^{4}=\left\{0,\frac{171}{250},\frac{93}{100},\frac{99}{100},1\right\}.$$

Then

$$\sin_{6/5,6/5}^{-1}(y_{1})=y_{1}\;{\;}_{2}\text{F}{\;}_{1}\left(\frac{5}{6},\frac{5}{6};\frac{11}{6};y_{1}^{\frac{6}{5}}\right)<1<\frac{\pi }{3}=\pi_{\frac{6}{5},\frac{6}{5}}x_{1}$$

and so

$$\sin_{6/5,6/5}(\pi_{6/5,6/5}x_{1})>y_{1}.$$

In addition,

$$\sin_{6/5,6/5}^{-1}(y_{2})<2<\pi_{6/5,6/5}x_{2}\;\text{and}\;\sin_{6/5,6/5}^{-1}(y_{3})<3<\pi_{6/5,6/5}x_{3},$$

so

$$\sin_{6/5,6/5}(\pi_{6/5,6/5}x_{j})>y_{j}\;j=2,3.$$

Let *p* be as in the hypothesis. Then, similar to the previous case (a),

6.1 $$\sin_{p,p}(\pi_{p,p}x_{j})>y_{j}\;j=1,2,3.$$

Set

$$\begin{array}{rl} \ell_{j}(x) & =\frac{y_{j+1}-y_{j}}{x_{j+1}-x_{j}}(x-x_{j})+y_{j}\;j=0,1,3\\ \ell_{2}(x) & =1. \end{array}$$

By strict concavity and (6.1),

$$\sin_{p,p}(\pi_{p,p}x)>\ell_{j}(x)\;\forall \;x\in (x_{j},x_{j+1})\;j=0,1,3.$$

Let

$$I_{j}=2\sqrt{2}\int_{x_{j}}^{x_{j+1}}\ell_{j}(x)\sin(5\pi x)\;dx\;j=0,1,2,3.$$

Then,

$$a_{5}(p,p)>\sum_{j=0}^{3}I_{j}>\frac{3}{100}>0$$

as claimed.

 *Bound* (c). Let *p* be as in the hypothesis. Set

$${\left\{x_{j}\right\}}_{j=0}^{5}=\left\{0,\frac{1}{14},\frac{1}{7},\frac{2}{7},\frac{3}{7},\frac{1}{2}\right\}\;\text{and}\;{\left\{y_{j}\right\}}_{j=0}^{5}=\left\{0,\frac{283}{500},\frac{106}{125},1,1,1\right\}.$$

Then

$$\sin_{6/5,6/5}^{-1}(y_{1})<\frac{73}{100}<\pi_{6/5,6/5}x_{1}\;\text{and}\;\sin_{6/5,6/5}^{-1}(y_{2})<\frac{147}{100}<\pi_{6/5,6/5}x_{2}.$$

Hence,

$$\sin_{p,p}(\pi_{p,p}x_{j})>y_{j}\;j=1,2.$$

Put

$$\begin{array}{rl} \ell_{j}(x) & =\frac{y_{j+1}-y_{j}}{x_{j+1}-x_{j}}(x-x_{j})+y_{j}\;j=0,1\\ \ell_{4}(x) & =1. \end{array}$$

Then,

$$\sin_{p,p}(\pi_{p,p}x)>\ell_{j}(x)\;\forall \;x\in (x_{j},x_{j+1})\;j=0,1.$$

Let

$$\begin{array}{rl} I_{j} & =2\sqrt{2}\int_{x_{j}}^{x_{j+1}}\ell_{j}(x)\sin(7\pi x)\;dx\;j=0,1,4\\ I_{j} & =2\sqrt{2}\int_{x_{j}}^{x_{j+1}}\sin_{p,p}(\pi_{p,p}x)\sin(7\pi x)\;dx\;j=2,3. \end{array}$$

Because sin⁡(7πx) is negative for *x*∈(*x*_2_,*x*_3_) and positive for *x*∈(*x*_3_,*x*_4_), then *I*_2_+*I*_3_>0. Hence,

$$a_{7}(p,p)>I_{0}+I_{1}+I_{4}>\frac{3}{1000}>0.$$

 *Bound* (d) Note that

$$\pi_{12/11,12/11}=\frac{11\pi \sqrt{2}}{3(\sqrt{3}-1)}.$$

Let *p* be as in the hypothesis. Set

$${\left\{x_{j}\right\}}_{j=0}^{5}=\left\{0,\frac{1}{18},\frac{1}{9},\frac{1}{3},\frac{4}{9},\frac{1}{2}\right\}\;\text{and}\;{\left\{y_{j}\right\}}_{j=0}^{5}=\left\{0,\frac{17}{24},\frac{15}{16},\frac{15}{16},\frac{15}{16},\frac{15}{16}\right\}.$$

Then,

$$\sin_{12/11,12/11}^{-1}(y_{1})<\frac{112}{100}<\pi_{12/11,12/11}x_{1}\;\text{and}\;\sin_{12/11,12/11}^{-1}(y_{2})<\frac{233}{100}<\pi_{12/11,12/11}x_{2}.$$

Hence,

$$\sin_{p,p}(\pi_{p,p}x_{j})>y_{j}\;j=1,2.$$

Put

$$\begin{array}{rl} \ell_{j}(x) & =\frac{y_{j+1}-y_{j}}{x_{j+1}-x_{j}}(x-x_{j})+y_{j}\;j=0,1\\ \ell_{3}(x) & =1\;\ell_{4}(x)=\frac{15}{16}. \end{array}$$

Then,

$$\sin_{p,p}(\pi_{p,p}x)>\ell_{j}(x)\;\forall \;x\in (x_{j},x_{j+1})\;j=0,1,4.$$

Let

$$\begin{array}{rl} I_{j} & =2\sqrt{2}\int_{x_{j}}^{x_{j+1}}\ell_{j}(x)\sin(9\pi x)\;dx\;j=0,1,3,4\\ I_{2} & =2\sqrt{2}\int_{x_{2}}^{x_{3}}\sin_{p,p}(\pi_{p,p}x)\sin(9\pi x)\;dx. \end{array}$$

Then, *I*_2_>0. Hence,

$$a_{9}(p,p)>I_{0}+I_{1}+I_{3}+I_{4}=2\sqrt{2}\left(\frac{23}{216\pi^{2}}-\frac{1}{72\pi }\right)>0.$$

 ▪

**Figure 2.**
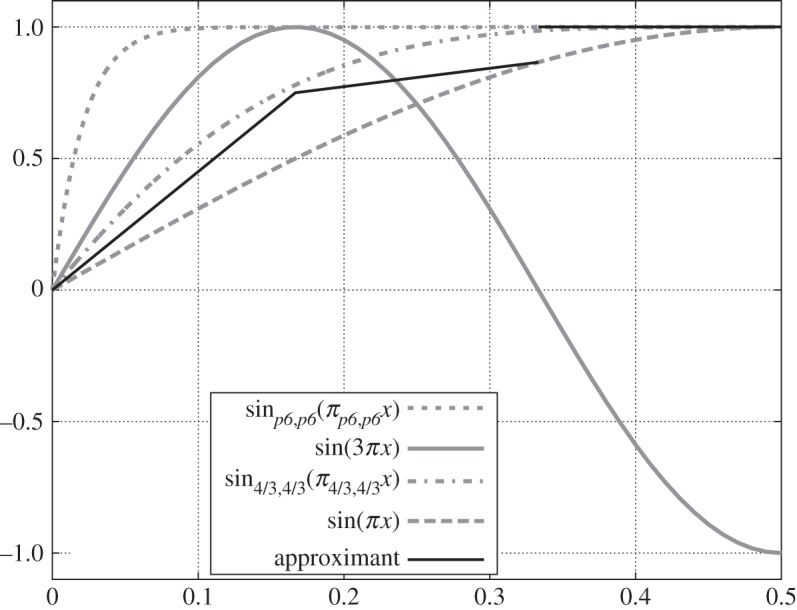
Approximants ℓ_*j*_(*x*) employed to show bound (a) in lemma 6.1. For reference, we also show $\sin_{p_{6},p_{6}}(\pi_{p_{6},p_{6}}x)$, sin⁡(3πx), $\sin_{4/3,4/3}(\pi_{4/3,4/3}x)$ and $\sin_{2,2}(\pi x)=\sin(\pi x)$.

Corollary 6.2 is a direct consequence of combining (a) and (d) from this lemma with theorem 5.1.

**Corollary 6.2**

*Set* *r*=2 *and suppose that* $1<{\tilde{p}}_{2}<\frac{12}{11}$ *is such that*

$$\sum_{j=3}^{\infty }|a_{j}({\tilde{p}}_{2},{\tilde{p}}_{2})|=a_{1}({\tilde{p}}_{2},{\tilde{p}}_{2}).$$

*There exists* *ε*>0 *such that* *A* *is invertible for all* $p\in ({\tilde{p}}_{2}-\varepsilon ,{\tilde{p}}_{2}+\varepsilon )$.

See figure 3.

**Figure 3.**
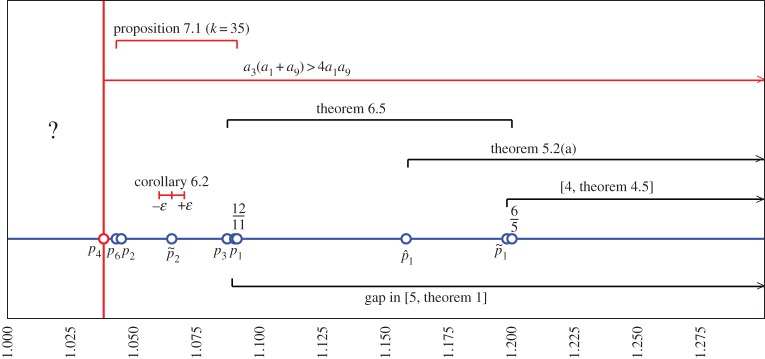
Relation between the various statements of this paper with those of references [4,5], for the case *p*=*q*. The positions of *p*_1_, ${\tilde{p}}_{2}$ and the value of *ε* are set only for illustration purposes, as we are certain only that $p_{2}<{\tilde{p}}_{2}<p_{3}$. (Online version in colour.)

**Remark 6.3**

In Binding *et al.* [5], it was claimed that the hypothesis of (1.2) held true whenever *p*=*q*≥*p*_1_ for a suitable $1<p_{1}<\frac{12}{11}$. The argument supporting this claim [5], §4 was separated into two cases: *p*≥2 and $\frac{12}{11}\leq p<2$. With our definition^3^ of the Fourier coefficients, in the latter case, it was claimed that |*a*_*j*_| was bounded above by

$$\frac{2\sqrt{2}\pi_{12/11,12/11}}{j^{2}\pi^{2}}{\left(\int_{0}^{1/2}\sin_{p,p}^{\prime \prime }{\left(\pi_{p,p}t\right)}^{2}\;dt\right)}^{1/2}{\left(\int_{0}^{1/2}\sin{\left(j\pi t\right)}^{2}\;dt\right)}^{1/2}.$$

As it turns, there is a missing power 2 in the term *π*_12/11,12/11_ for this claim to be true. This corresponds to taking second derivatives of $\sin_{p,p}(\pi_{p,p}t)$ and it can be seen by applying the Cauchy–Schwartz inequality in (5.1). The missing factor is crucial in the argument and renders the proof of Binding *et al*. [5], theorem 1 incomplete in the latter case.

In the paper [4], published a few years later, it was claimed that the hypothesis of (1.2) held true for $p=q\geq {\tilde{p}}_{1}$, where ${\tilde{p}}_{1}$ is defined by (1.6). It was then claimed that an approximated solution of (1.6) was near $1.05<\frac{12}{11}$. An accurate numerical approximation of (1.6), based on analytical bounds on *a*_1_(*p*,*p*), give the correct digits ${\tilde{p}}_{1}\approx 1.198236>\frac{12}{11}$. Therefore, neither the results of Binding *et al*. [5] nor those of Bushell & Edmunds [4] include a complete proof of invertibility of the change of coordinates in a neighbourhood of $p=\frac{12}{11}$.

Accurate numerical estimation of *a*_1_(*p*,*p*) shows that the identity (5.4) is valid as long as $p>{\hat{p}}_{1}\approx 1.158739>\frac{12}{11}$, which improves slightly upon the value ${\tilde{p}}_{1}$ from [4]. However, as remarked in [4], the upper bound,

$$|a_{j}|\leq \frac{2\sqrt{2}\pi_{p,p}}{j^{2}\pi^{2}}$$

ensuring (5.5) and hence the validity of theorem 5.2(a), is too crude for small values of *p*. Note, for example, that the correct regime is $a_{j}(p,p)\to 2\sqrt{2}/j\pi ,$ whereas $\pi_{p,p}\to \infty$ as *p*→1 (see appendix A). Therefore, in order to determine invertibility of *A* in the vicinity of $p=q=\frac{12}{11}$, it is necessary to find sharper bounds for the first few terms |*a*_*j*_|, and employ (1.2) directly. This is the purpose of the next lemma.

**Lemma 6.4**

*Let* $1<p\leq \frac{6}{5}$. *Then*,

(a) $a_{1}(p,p)>\frac{839}{1000}$.
(b) $a_{3}(p,p)<\frac{151}{500}$.
(c) $a_{5}(p,p)<\frac{181}{1000}$.
(d) $a_{7}(p,p)<\frac{13}{100}$.

**Proof.**

We proceed in a similar way as in the proof of lemma 6.1. Let *p* be as in the hypothesis.

 *Bound* (a). Set

$${\left\{x_{j}\right\}}_{j=0}^{3}=\left\{0,\frac{31}{250},\frac{101}{500},\frac{1}{2}\right\}\;\text{and}\;{\left\{y_{j}\right\}}_{j=0}^{5}=\left\{0,\frac{4}{5},\frac{19}{20},1\right\}.$$

Then,

$$\sin_{6/5,6/5}^{-1}(y_{1})<\frac{129}{100}<\pi_{6/5,6/5}x_{1}\;\text{and}\;\sin_{6/5,6/6}^{-1}(y_{2})<\frac{211}{100}<\pi_{6/5,6/5}x_{2}$$

and so

$$\sin_{p,p}(\pi_{p,p}x_{j})>y_{j}\;j=1,2.$$

Let

$$\begin{array}{rl} \ell_{j}(x) & =\frac{y_{j+1}-y_{j}}{x_{j+1}-x_{j}}(x-x_{j})+y_{j}\\ I_{j} & =2\sqrt{2}\int_{x_{j}}^{x_{j+1}}\ell_{j}(x)\sin(\pi x)\;dx \end{array}\}\;j=0,1,2.$$

Then,

$$\sin_{p,p}(\pi_{p,p}x)>\ell_{j}(x)\;\forall \;x\in (x_{j},x_{j+1})\;j=0,1,2.$$

Hence,

$$a_{1}(p,p)>I_{0}+I_{1}+I_{2}>\frac{839}{1000}.$$

 *Bound* (b). Set

$${\left\{x_{j}\right\}}_{j=0}^{2}=\left\{0,\frac{1}{3},\frac{1}{2}\right\}\;\text{and}\;{\left\{y_{j}\right\}}_{j=0}^{2}=\left\{0,\frac{99}{100},1\right\}.$$

Then,

$$\sin_{6/5,6/5}^{-1}(y_{1})<3<\pi_{6/5,6/5}x_{1}\;\text{and so}\;\sin_{p,p}(\pi_{p,p}x_{1})>y_{1}.$$

Let

$$\begin{array}{rl} \ell_{0}(x) & =1\\ \ell_{1}(x) & =\frac{y_{2}-y_{1}}{x_{2}-x_{1}}(x-x_{1})+y_{1}\\ I_{j} & =2\sqrt{2}\int_{x_{j}}^{x_{j+1}}\ell_{j}(x)\sin(3\pi x)\;dx\;j=0,1. \end{array}$$

Then,

$$\sin_{p,p}(\pi_{p,p}x)>\ell_{1}(x)\;\forall \;x\in (x_{1},x_{2})$$

and hence

$$a_{3}(p,p)<I_{0}+I_{1}<\frac{151}{500}.$$

 *Bound* (c). Set

$${\left\{x_{j}\right\}}_{j=0}^{2}=\left\{0,\frac{1}{5},\frac{2}{5},\frac{1}{2}\right\}$$

and let

$$\begin{array}{rl} I_{j} & =2\sqrt{2}\int_{x_{j}}^{x_{j+1}}\sin_{p,p}(\pi_{p,p}x)\sin(5\pi x)\;dx\;j=0,1\\ I_{2} & =2\sqrt{2}\int_{x_{2}}^{x_{3}}\sin(5\pi x)\;dx. \end{array}$$

Then, *I*_0_+*I*_1_<0, so

$$a_{5}(p,p)<I_{2}=\frac{2\sqrt{2}}{5\pi }<\frac{181}{1000}.$$

 *Bound* (d). Set

$${\left\{x_{j}\right\}}_{j=0}^{4}=\left\{0,\frac{1}{7},\frac{2}{7},\frac{5}{14},\frac{3}{7},\frac{1}{2}\right\}$$

and

$$\begin{array}{rl} I_{j} & =2\sqrt{2}\int_{x_{j}}^{x_{j+1}}\sin_{p,p}(\pi_{p,p}x)\sin(7\pi x)\;dx\;j=0,1,3,4.\\ I_{2} & =2\sqrt{2}\int_{x_{2}}^{x_{3}}\sin(7\pi x)\;dx \end{array}$$

Then, *I*_0_+*I*_1_<0 and *I*_3_+*I*_4_<0, so

$$a_{7}(p,p)<I_{2}=\frac{2\sqrt{2}}{7\pi }<\frac{13}{100}.$$

 ▪

The following result fixes the proof of the claim made in [5], §4 and claim 2 and improves the threshold of invertibility determined in [4], theorem 4.5.

**Theorem 6.5**

*There exists* $1<p_{3}<\frac{6}{5}$ *such that*

6.2 $$\pi_{p,p}<\frac{[a_{1}(p,p)-a_{3}(p,p)-a_{5}(p,p)-a_{7}(p,p)]\pi^{2}}{2\sqrt{2}(\pi^{2}/8-1-1/9-1/25-1/49)}\;\forall \;p\in \left(p_{3},\frac{6}{5}\right).$$

*The family* S *is a Schauder basis of L*^*r*^(*0,1*) *for all* $p_{3}<p=q<\frac{6}{5}$ *and r>1.*

**Proof.**

Both sides of (6.2) are continuous functions of the parameter *p*>1. The right-hand side is bounded. The left-hand side is decreasing as *p* increases and $\pi_{p,p}\to \infty$ as *p*→1. By virtue of lemma 6.4,

$$\pi_{6/5,6/5}=\frac{10\pi }{3}<12<\frac{(a_{1}(6/5,6/5)-a_{3}(6/5,6/5)-a_{5}(6/5,6/5)-a_{7}(6/5,6/5))\pi^{2}}{2\sqrt{2}(\pi^{2}/8-1-1/9-1/25-1/49)}.$$

Hence, the first statement is ensured as a consequence of the intermediate value theorem.

From (5.1), it follows that

$$\sum_{j\notin \{1,3,5,7\}}|a_{j}(p,p)|<\frac{2\sqrt{2}\pi_{p,p}}{\pi^{2}}\left(\frac{\pi^{2}}{8}-1-\frac{1}{9}-\frac{1}{25}-\frac{1}{49}\right)$$

for all $p_{3}<p<\frac{6}{5}$. Lemma 6.1 guarantees positivity of *a*_*j*_ for *j*=3,5,7. Then, by rearranging this inequality, the second statement becomes a direct consequence of (1.2). ▪

A sharp numerical approximation of the solution of the equation with equality in (6.2) gives $p_{3}\approx 1.087063<\frac{12}{11}$ (figure 3).

## 7. The thresholds for invertibility and the regions of improvement

If sharp bounds on the first few Fourier coefficients *a*_*j*_(*p*,*q*) are at hand, the approach employed above for the proof of theorem 6.5 can also be combined with the criteria (4.1) or (4.2). A natural question is whether this would lead to a positive answer to the question of invertibility for *A*, whenever

$$\sum_{k=3}^{\infty }a_{j}\geq a_{1}.$$

In the case of (4.1), we see below that this is indeed the case. The key statement is summarized as follows.

**Proposition 7.1**

*Let* *r*=2 *and* 5≤*k*≢_2_0. *Suppose that*

(a) *a*_3_>0, *a*_9_>0 and *a*_*j*_≥0 for all other 5≤*j*≤*k*.
(b) *a*_3_(*a*_1_+*a*_9_)>4*a*_9_*a*_1_.

*If*

7.1 $$\pi_{p,q}<\left(a_{1}+a_{9}-\sum_{\begin{array}{c} 3\leq j\leq k\\ j\notin \{1,9\} \end{array}}a_{j}\right)\frac{\pi^{2}}{2\sqrt{2}(\pi^{2}/8-\sum_{\begin{array}{c} 1\leq j\leq k\\ j{≢}_{2}0 \end{array}}^{k}(1/j^{2}))},$$

*then* *A* *is invertible*.

**Proof.**

Assume that the hypotheses are satisfied. The combination of (5.1) and (7.1) gives

$$\sum_{j=k+1}^{\infty }|a_{j}|\leq \frac{2\sqrt{2}\pi_{p,q}}{\pi^{2}}\left(\frac{\pi^{2}}{8}-\sum_{\begin{array}{c} 1\leq j\leq k\\ j{≢}_{2}0 \end{array}}^{k}\frac{1}{j^{2}}\right)<a_{1}+a_{9}-\sum_{\begin{array}{c} 3\leq j\leq k\\ j\notin \{1,9\} \end{array}}a_{j}.$$

Then,

$$\sum_{j\notin \{1,9\}}|a_{j}|=\sum_{\begin{array}{c} 3\leq j\leq k\\ j\notin \{1,9\} \end{array}}a_{j}+\sum_{\begin{array}{c} j>k\\ j\notin \{1,9\} \end{array}}|a_{j}|<a_{1}+a_{9}$$

and so the conclusion follows from (4.1). ▪

We now discuss the connection between the different statements established in the previous sections with those of the papers [3–5]. For this purpose, we consider various accurate approximations of *a*_*j*_ and $\sum a_{j}$. These approximations are based on the next explicit formulae

$$\pi_{p,q}=\frac{2B(1/q,(p-1)/p)}{q}=\frac{2\Gamma ((p-1)/p)\Gamma (1/q)}{q\Gamma ((p-1)/p+1/q)}$$

and

$$\begin{array}{rl} a_{j}(p,q) & =\frac{2\sqrt{2}}{j\pi }\int_{0}^{1}\cos\left(\frac{j\pi x}{\pi_{p,q}}\;{\;}_{2}\text{F}{\;}_{1}\left(\frac{1}{p},\frac{1}{q};1+\frac{1}{q};x^{q}\right)\right)dx\\ & =\frac{2\sqrt{2}}{j\pi }\int_{0}^{1}\cos\left(\frac{j\pi }{2}I\left(\frac{1}{q},\frac{p-1}{p};x^{q}\right)\right)dx. \end{array}$$

Here, I is the incomplete beta function, *B* is the beta function and *Γ* is the gamma function. Moreover, by considering exactly the steps described in [4] for the proof of Bushell & Edmunds [4], (4.15), it follows that

$$\begin{array}{rl} \sum_{j=1}^{\infty }a_{j}(p,q) & =\frac{\sqrt{2}}{\pi }\int_{0}^{1}\log\left[\cot\left(\frac{\pi x}{2\pi_{p,q}}\;{\;}_{2}\text{F}{\;}_{1}\left(\frac{1}{p},\frac{1}{q};1+\frac{1}{q};x^{q}\right)\right)\right]dx\\ & =\frac{\sqrt{2}}{\pi }\int_{0}^{1}\log\left[\cot\left(\frac{\pi }{4}\;I\left(\frac{1}{q},\frac{p-1}{p};x^{q}\right)\right)\right]dx. \end{array}$$

Let us begin with the case of equal indices (figure 3). As mentioned in the Introduction,

$$\sum_{k=3}^{\infty }a_{j}(p_{2},p_{2})=a_{1}(p_{2},p_{2})$$

for *p*_2_≈1.043989. The condition *a*_3_(*p*,*p*)(*a*_1_(*p*,*p*)+*a*_9_(*p*,*p*))>4*a*_9_(*p*,*p*)*a*_1_(*p*,*p*) is fulfilled for all $p_{4}<p<\frac{12}{11}$, where *p*_4_≈1.038537. The Fourier coefficients *a*_*j*_(*p*,*p*)≥0 for all 1≤*j*≤35 whenever $1<p<\frac{12}{11}$. Remarkably, we need to get to *k*=35, for a numerical verification of the conditions of proposition 7.1 allowing *p*<*p*_2_. Indeed, we remark the following.

(a) For *k*=3,…,33, the condition (7.1) hold true only for $p_{5}<p<\frac{12}{11}$, where *p*_5_≥1.044573>*p*_2_.
(b) For *k*=35, the condition (7.1) does hold true for $p_{6}<p<\frac{12}{11}$, where *p*_6_≈1.043917<*p*_2_.

This indicates that the threshold for invertibility of *A* in the Hilbert space setting for *p*=*q* is at least *p*_6_.

**Figure 4.**
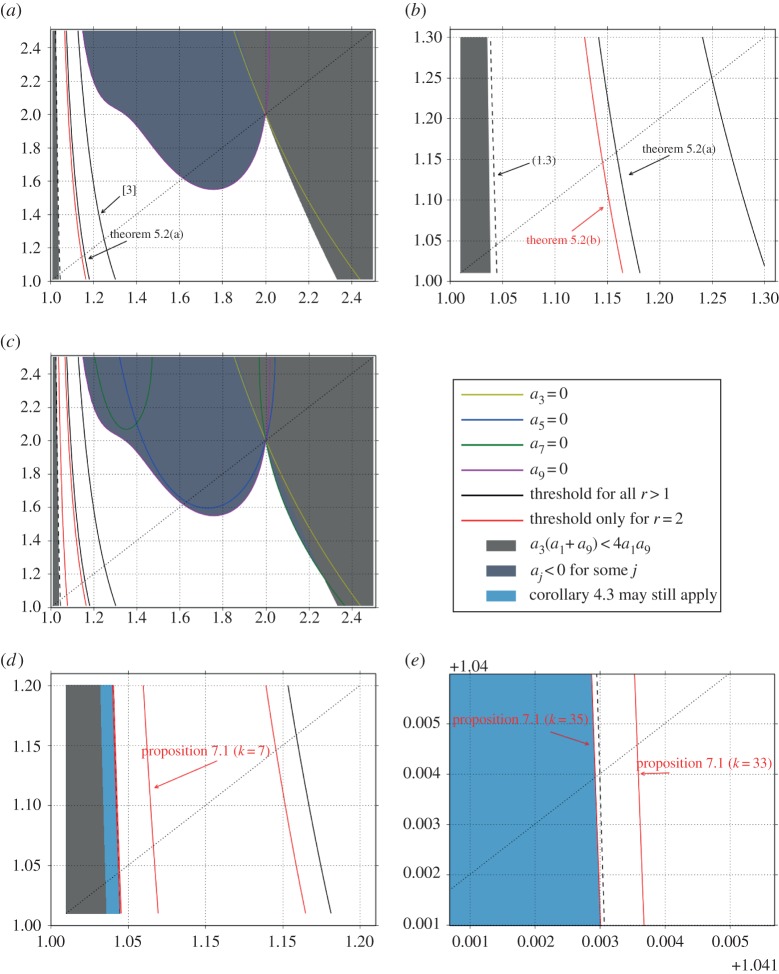
(*a*–*e*) Different relations and boundaries between the regions of the (*p*,*q*)-plane where theorem 5.2(a) and (b) as well as proposition 7.1 (with different values of *k*) apply. In all graphs, *p* corresponds to the horizontal axis and *q* to the vertical axis and the dotted line shows *p*=*q*. (Online version in colour.)

**Figure 5.**
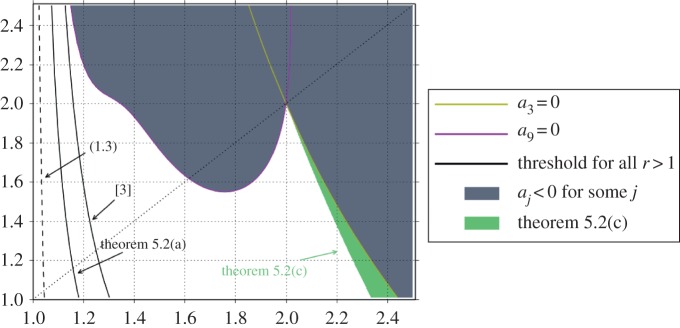
Region of the (*p*,*q*)-plane where theorem 5.2(c) applies. Even when we know *A* is invertible in this region as a consequence of theorem 5.2(a), the upper bound on the Riesz constant provided by (4.2) improves upon that provided by (1.2) (case *r*=2). In this graph, *p* corresponds to the horizontal axis and *q* to the vertical axis and the dotted line shows *p*=*q*. (Online version in colour.)

Now, we examine the general case. The graphs shown in figures 4 and 5] correspond to regions in the (*p*,*q*)-plane near (*p*,*q*)=(1,1). Curves on figure 4 that are in red (online version) are relevant only to the Hilbert space setting *r*=2. Black curves (online version) pertain to *r*>1.

 Figure 4*a* and a blowup shown in figure 4*b* have two solid (black) lines. One that shows the limit of applicability of theorem 5.2(a) and one that shows the limit of applicability of the result of [3]. The dashed line indicates where (1.3) occurs. To the left of that curve, (1.2) is not applicable. There are two filled regions of different colours in (figure 4*a*), which indicate where *a*_3_(*a*_1_+*a*_9_)<4*a*_1_*a*_9_ and where *a*_*j*_<0 for *j*=3,9. Proposition 7.1 is not applicable in the union of these regions. We also show the lines where *a*_3_=0 and *a*_9_=0. The latter forms part of the boundary of this union. The solid red line corresponding to the limit of applicability of theorem 5.2(b) is also included in figure 4*a*,*d*. To the right of that line, in the white area, we know that *A* is invertible for *r*=2. The blowup in figure 4*b* clearly shows the gap between theorem 5.2(a),(b) in this *r*=2 setting.

Certainly, *p*=*q*=2 is a point of intersection for all curves where *a*_*j*_=0 for *j*>1. These curves are shown in figure 4*c* also for *j*=5 and *j*=7. In this figure, we also include the boundary of the region where *a*_3_(*a*_1_+*a*_9_)<4*a*_1_*a*_9_ and the region where *a*_*j*_<0 now for *j*=3,5,7,9. Note that the curves for *a*_7_=0 and *a*_9_=0 form part of the boundary of the latter. Comparing figure 4*a* and figure 4*c*, the new line that cuts the *p* axis at *p*≈1.1 corresponds to the limit of where proposition 7.1 for *k*=7 is applicable (for *p* to the right of this line). The gap between the two red lines (case *r*=2) indicates that proposition 7.1 can significantly improve the threshold for basisness with respect to a direct application of theorem 5.2(b).

As we increase *k*, the boundary of the corresponding region moves to the left, see the blowups in figure 4*d*,*e*. The two further curves in red located very close to the vertical axis, correspond to the precise value of the parameter *k* where proposition 7.1 allows a proof of invertibility for the change of coordinates which includes the break made by (1.3). For *k*<35, the region does not include the dashed black line, for *k*=35, it does include this line. The region shown inQ1 blue indicates a possible place where corollary 4.3 may still apply, but further investigation in this respect is needed.

Figure 5 concerns the statement of theorem 5.2(c). The small wedge shown in green is the only place where the former is applicable. As it turns, it appears that the conditions of corollary 4.4 prevent it to be useful for determining invertibility of *A* in a neighbourhood of (*p*,*q*)=(1,1). However, in the region shown in green, the upper bound on the Riesz constant consequence of (4.2) is sharper than that obtained from (1.2).

## Footnotes

1 See http://arxiv.org/abs/1405.7337.

2 See also [5], lemma 5.

3 The Fourier coefficients in Binding *et al*. [5] differ from *a*_*j*_(*p*,*p*) by a factor of $\sqrt{2}$. Note that the ground eigenfunction of the *p*-Laplacian equation in Binding *et al*. [5] is denoted by *S*_*p*_(*x*) and it equals $\sin_{p,p}(x)$ as defined above. A key observation here is the *p*-Pythagorean identity $|\sin_{p,p}(x){|}^{p}+|\sin_{p,p}^{\prime }(x){|}^{p}=1=|S_{p}(x){|}^{p}+|S_{p}^{\prime }(x){|}^{p}$.

## Data accessibility

All the numerical quantities reported in this paper are accurate up to the last digit shown, which is rounded to the nearest integer. In the online version available at http://arxiv.org/abs/1405.7337, we have included fully reproducible computer codes which can be employed to verify the calculations reported.

## Appendix A. The shape of $\sin_{p,p}$ as *p*→1

Part of the difficulties for a proof of basisness for the family S in the regime *p*=*q*→1 has to do the fact that the Fourier coefficients of *s*_1_ approach those of the function sgn(sin⁡(πx)). In this appendix, we show that, indeed

A 1 $$\lim_{p\to 1}\left(\max_{0\leq x\leq 1}|s_{1}(x)-\mathrm{sgn}(\sin(\pi x))|\right)=0.$$

Proof. Note that

$$\frac{d^{n}}{dy^{n}}s_{1}^{-1}(y)>0\;\forall \;0<y<1,\;n=0,1,2.$$

Let *y*_1_(*p*)∈(0,1) be the (unique) value, such that

$$\frac{d}{dy}s_{1}^{-1}(y_{1}(p))=\frac{1}{{\left(1-y_{1}{\left(p\right)}^{p}\right)}^{1/p}}=\pi_{p,p}.$$

Then,

$$y_{1}(p)={\left(1-\frac{1}{\pi_{p,p}^{p}}\right)}^{1/p}\to 1\;p\to 1.$$

Let

$$h(t)=1-\frac{t}{y_{1}(p)}$$

be the line passing through the points (0,1) and (*y*_1_(*p*),0). There exists a unique value *y*_2_(*p*)∈(0,*y*_1_(*p*)) such that

$$\frac{1}{\pi_{p,p}}\frac{1}{{\left(1-y_{2}{\left(p\right)}^{p}\right)}^{1/p}}=h(y_{2}(p)).$$

This value is unique because of monotonicity of both sides of this equality, and it exists by bisection. As all the functions involved are continuous in *p*, then also *y*_2_(*p*) is continuous in the parameter *p*. Moreover,

$$y_{2}(p)\to 1\;p\to 1.$$

Indeed, by clearing the equation defining *y*_2_(*p*), we obtain

$${\left(1-\frac{y_{2}(p)}{y_{1}(p)}\right)}^{p}(1-y_{2}{\left(p\right)}^{p})=\frac{1}{\pi_{p,p}^{p}}.$$

The right-hand side, and thus the left-hand side, approach 0 as *p*→1. Then, one (and hence both) of the two terms multiplying on the left should approach 0. Let $P_{p}$ be the polygon which has as vertices (ordered clockwise)

$$\begin{array}{rl} v_{1}(p) & =\left(0,\frac{1}{\pi_{p,p}}\right),\;v_{2}(p)=(y_{2}(p),h(y_{2}(p))),\\ v_{3}(p) & =(y_{1}(p),1),\;v_{4}(p)=(y_{1}(p),0)\;\text{and}\;v_{5}=(0,0). \end{array}$$

As

$$\begin{array}{rl} v_{1}(p) & \to (0,0)=v_{5},\;v_{2}(p)\to (1,0),\\ v_{3}(p) & \to (1,1)\;\text{and}\;v_{4}(p)\to (1,0); \end{array}$$

$B_{p}\to ([0,1]\times \{0\})\cup (\{1\}\times [0,1])$ in Hausdorff distance. Then, the area of $P_{p}$ approaches 0 as *p*→1. Moreover, $P_{p}$ covers the graph of

$$\frac{1}{\pi_{p,p}}\frac{1}{{\left(1-t^{p}\right)}^{1/p}}$$

for 0<*t*<*y*_1_(*p*). Thus,

$$x_{1}(p)=s_{1}^{-1}(y_{1}(p))=\frac{1}{\pi_{p,p}}\int_{0}^{y_{1}(p)}\frac{dt}{{\left(1-t^{p}\right)}^{1/p}}\to 0\;p\to 1.$$

Hence, there is a point (*x*_1_(*p*),*y*_1_(*p*)) on the graph of *s*_1_(*x*) such that $0<x_{1}(p)<\frac{1}{2}$ and

$$(x_{1}(p),y_{1}(p))\to (0,1).$$

The proof of (.1) is completed from the fact that, as *s*_1_(*x*) is concave (because its inverse function is convex), the piecewise linear interpolant of *s*_1_(*x*) for the family of nodes $\left\{0,x_{1}(p),\frac{1}{2}\right\}$ has a graph below that of *s*_1_(*x*). ▪
